# Migrating hoverflies as potential food source for co-migrating insectivorous birds

**DOI:** 10.1098/rsos.241743

**Published:** 2025-03-05

**Authors:** Antonín Hlaváček, Peter Mikula, Jiří Hadrava, Radek K. Lučan

**Affiliations:** ^1^ Department of Zoology, Faculty of Science, Charles University, Viničná 7, Prague 2 12800, Czechia; ^2^ Department of Ecology, Faculty of Environmental Sciences, Czech University of Life Sciences Prague, Kamýcká 129, Prague 16500, Czechia; ^3^ TUM School of Life Sciences, Ecoclimatology, Technical University of Munich, Hans-Carl-von-Carlowitz-Platz 2, 85354 Freising, Germany; ^4^ Institute for Advanced Study, Technical University of Munich, Lichtenbergstraße 2a, 85748 Garching, Germany

**Keywords:** co-migration, Diptera, mountain barrier, predator, prey, Syrphidae

## Abstract

Most migrating birds must replenish energy reserves during migration. Food availability significantly influences migratory routes and can even force migrants to detour, but still little is known about potential co-migration between insectivorous birds and their insect prey. To address this gap, we focused on day-flying insects and the insectivorous birds migrating through the Červenohorské sedlo mountain pass, Czech Republic. During four seasons of insect and bird trapping, using Malaise trap and mist-nets, respectively, we recorded 23 094 birds of 80 species and 35 087 migrating hoverflies (Syrphidae) of 47 species. We found a strong temporal correlation between the number of migrating hoverflies and insectivorous birds crossing the mountain pass. The observed pattern suggests that a similar phenomenon may occur in lowlands, where both groups stop over before and after crossing the mountains. These stopovers may provide migratory birds with abundant and reliable food resources. We also found that hoverflies comprised 88% of the biomass of all trapped insects, making them the most abundant potential prey of migrating birds. Our results outline the co-migration of birds and hoverflies and shed light on possible predator–prey dynamics during migration.

## 1. Introduction

Billions of animals across the globe migrate annually between distant locations [1–4]. Migration leads to a temporal change of habitat in response to unfavourable conditions, usually in search of food and breeding opportunities [5,6]. This specific behaviour has evolved in most lineages of metazoans [6], and it is no surprise that most migrating groups are formed by a mixture of species from various phyla. Temporal and spatial co-occurrence of migrating species could easily be explained as a common adaptive response to abiotic drivers, such as geographical boundaries, weather conditions or seasonal dynamics [7–9]. The co-occurrence of diverse assemblages of migratory animals may result in interspecific interactions between migrants, particularly at the predator–prey level. These interactions have recently attracted strong interest among ecologists considering food chain stability, climate change or phenological mismatch [9–11].

The need to replenish fuel reserves during migration shapes migratory routes [12–14], number of stopover sites [15], defines migration distance [16] or its timing [17]. Based on optimal migration theory [18,19], predators should trade-off between fuel load and a number of refuelling stopovers to maximize their flight speed, minimize predation risks and avoid unfavourable conditions [19,20]. For migratory predators, it may be advantageous to follow spatial and temporal patterns in their prey migrations; predator–prey coupling can take different forms, from tracking or simultaneously migrating [21] to detouring from typical migratory routes [10]. For example, birds should balance between flights with stopovers, where they intensively forage before the next endurance flight, and fly-and-forage strategy, when they also cover some of the migration distance, but at reduced flight speed [14,22,23]. Both refuelling strategies are well known in many species; however, we usually lack data on the specific prey that migrating birds rely upon migration.

In insectivorous migratory birds, migratory flying insects may play this role, including hoverflies (Diptera: Syrphidae), common insect pollinators which can be found on all continents except Antarctica. For decades, hoverfly migration was considered to be rather a marginal issue; however, recent focus on that topic brought evidence of the annual migration of billions of hoverflies [24,25], outlining their crucial role in biomass transfer, pest control and pollination [24,26,27]. Hoverflies are, despite their sometimes faultless mimicry [28–30], a common part of the diet of many birds, including European species such as reed warblers [31], willow flycatchers [32], house martins [33] and others [34].

Here, we present 4 years of observations of bird and hoverfly autumnal migration at Červenohorské sedlo mountain pass, Czech Republic, and test whether the migration of birds and hoverflies is temporally synchronized while controlling for the potential effects of weather conditions. We also compare the biomass of hoverflies with other diurnal flying insects caught during migration.

## 2. Material and methods

### 2.1. Study site

**Figure 1.**
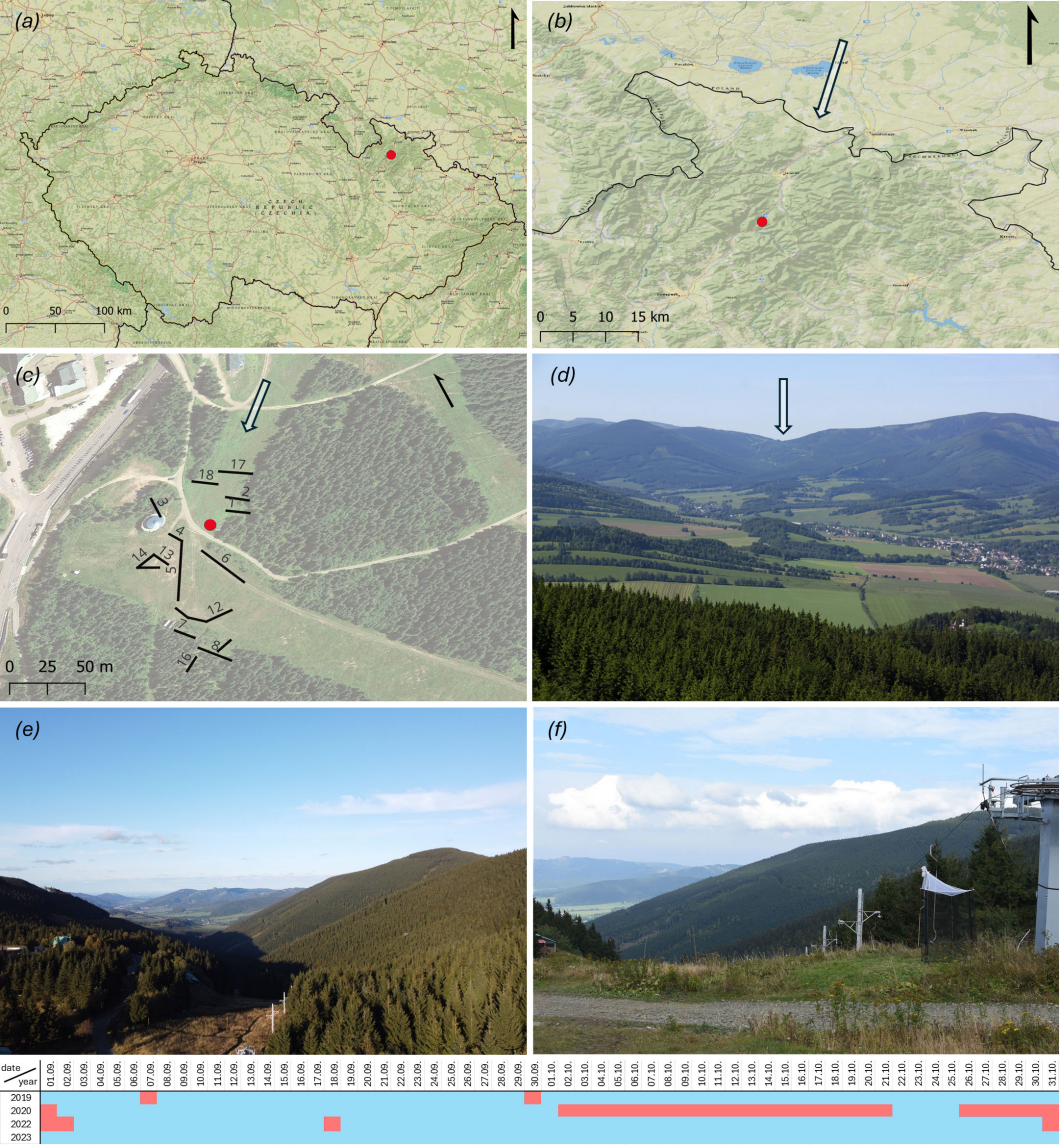
Collecting site and traps. North arrows are shown in black; presumed migratory routes are indicated by white arrows. (*a*) Location of Červenohorské sedlo, Czech Republic. (*b*) Detailed map of the Jeseníky mountains, with the study site marked by a red dot. (*c*) Close-up map of the collection site, red dot indicating the location of the Malaise trap, black lines representing mist-nets (for mesh size, see electronic supplementary material, data). (*d*) View of Červenohorské sedlo from the Polish side (photo by Martin Vavřík). (*e*) Aerial view of the migratory corridor directly from the collection site (drone photo by Tomáš Pospíšil). (*f*) Photograph of the collection site with the Malaise trap (photo by Jiří Hadrava). The timetable illustrates the catching periods during which both birds and hoverflies were monitored.

The study was conducted at the Červenohorské sedlo mountain pass (50.1245331 N, 17.1537733 E) in the northeast of the Czech Republic. Due to its position in a long mountain chain located perpendicularly to the prevailing orientation of flying migratory organisms, the site is a well-known migratory corridor, channelling autumn migration of insects, birds and bats to a narrow space of *ca* 400 m, most of which is covered by ski slopes facing to the north [35,36] (see figure 1).

### 2.2. Syrphidae collecting and identification and biomass weighting

In 2019 and 2020, two single-side blocked Malaise traps were placed on the edge of the ski slope (1020 m.a.s.l.), oriented to the north. We used two single-side blocked Malaise traps to distinguish between southward and northward migration. Since the pivotal study [35] has shown that autumn migration of hoverflies takes place from the beginning of September until the end of October and is minimally biased by local individuals (flying northward), we used only one single-side blocked Malaise trap (catching only hoverflies flying southward) in 2022 and 2023.

In 2019, the traps operated from 4 August to 31 October; in 2020, from 2 August to 2 October and from 22 to 25 October (the gap was caused by a storm which destroyed our traps); in 2022, from 3 September to 31 October; and in 2023, from 15 August to 1 November, depending on weather conditions (see table in figure 1). Traps were emptied daily after sunset. Traps of different sizes were used in 2019 and 2020 (lower side height = 1 m, higher side height = 2 m, width = 1.3 m, *A* = 1.95 m^2^) and in 2022 and 2023 (lower side height = 2 m, higher side height = 2.5 m, width = 1.3 m, *A* = 5.2 m^2^) [35]. We analysed data only from the peak migration period (1 September–30 October).

To assess the biomass of day-migrating insects, all trapped insect specimens from 2022 were sorted into orders; Diptera were additionally sorted into hoverflies and the rest. We then weighted their biomass using Kern PEJ precision balances (*e* = 0.01 g, *d* = 0.001 g), following Hallman’s wet biomass weighting protocol [37].

Hoverflies from 2019 and 2020 were pinned, while those trapped in 2022 and 2023 are stored in denatured alcohol; all are kept in A.H.’s private collection. All specimens were identified to the species level using up-to-date identification keys [38–40].

### 2.3. Bird trapping and identification

Data on the bird migration intensity at the locality were collected using standardized methodology introduced in the study site already in 2015 [36]. Migrating birds (>95% were small passerines) were captured daily (except days with harsh weather) using mist-nets (Ecotone, Poland) installed from mid-August to mid-November along and across the ski-slope at the top of the mountain pass, i.e. at the place where most migrating birds cross the pass. The total length of mist-nets was 394 m, and the mesh size used was 16 mm (286 m), 22 mm (54 m), 30 mm (18 m), and 60 mm (36 m). Mist-nets were installed at the same position each year. The capture efficiency of elusive insectivorous species (genera *Sylvia*, *Phylloscopus*, *Troglodytes*, *Prunella*, *Regulus*, *Acrocephalus*, *Locustella*, *Anthus* and *Parus*) was enhanced by playback of their voice mix reproduced from four loudspeakers permanently installed close to the open mist-nets, using the same avian voice mix for each period across years to make research efforts comparable. Birds were captured during day and night, but only data on daily captures have been used for the analysis, since hoverflies migrate exclusively during the day. Captured birds were processed using standard protocol for bird-ringing studies [41].

Except for mist-netting, the intensity of diurnal bird migration was monitored using visual observation of birds crossing the site from the north to the south. Each day, experienced birders estimate the order of magnitude at which birds were migrating over the mountain pass. Since exact census of each single bird is impossible due to the flocking behaviour of many species (particularly Fringillidae, Turdidae and Sturnidae), we recorded the intensity of migration based on visual estimation of bird numbers, where counts were rounded to the nearest values on a semi-quantitative scale (0, 10, 50, 100, 200, 500, 1000, 2000, 5000, 10 000, 20 000, 50 000, 100 000, 500 000).

### 2.4. Weather conditions

Temperature (°C), wind speed (m s^−1^), precipitation (mm d^−1^) and relative barometric pressure (hPa) were retrieved from a HadexWH 1080 weather station, placed in the proximity (*ca* 100 m) of the traps. Daily mean, maximum and minimum of these weather variables were calculated from data obtained between 06.00 and 18.00 (UTC+2.00).

### 2.5. Statistical analysis

First, we checked the distribution of predictor variables and their mutual collinearity, revealing generally low degree of multi-collinearity (electronic supplementary material, figure S1). However, we found a strong correlation between the size of Malaise traps used to monitor hoverflies and year (*r*_spearman_ = 0.95); hence, we excluded trap size from further analyses and used only year as a predictor variable. During exploratory analysis, we also found that temperature was relatively strongly correlated with both date (*r*_spearman_ = −0.45) and precipitations (*r*_spearman_ = −0.54). Hence, we included in our models only date and precipitations. For analysis, we used only data from days when we had complete data for birds and hoverflies (see figure 1). Then, we tested for the association between the number of caught birds in mist-nets (response variable) as a function of the number of caught hoverflies (log_10_-transformed + 1), second polynomial of Julian date, year (used as a categorical variable), wind speed and precipitations (log_10_-transformed + 0.1) (predictors) using a linear model with a negative binomial distribution (because of high overdispersion) using the function glm.nb in the MASS v. 7.3-55 package [42]. To test the robustness of the results (with qualitatively similar results indicating that model outputs are not model type-dependent), we also re-ran this model but using a linear model with Gaussian distribution (response variable, i.e. number of caught birds, was log_10_-transformed + 1 here). Model assumptions were visually checked using diagnostic graphs via the plot function. In all models, we again checked the collinearity among predictors by estimating variation inflation factors (VIFs) using the vif function in the car v. 3.0-12 package [43]. In general, VIF > 4 may indicate problematic collinearity [44]; we detected VIFs < 1.55 in all cases. Finally, we calculated Nagelkerke’s pseudo-*R* ^2^ of the negative binomial model using the model_performance function in the performance v. 0.12.3 package [45]; variance explained by the Gaussian model was provided in the model summary. Because all models produced qualitatively similar results, we report only the results of the first model in the main text (i.e. the number of birds caught in mist-nets as a response variable); results of the second are provided in electronic supplementary material, table S1. To test if bird capture data reflect the observed migration intensity, we used Spearman’s rank correlation rho, where daily totals of captured birds were tested against daily estimates of all migrating birds observed visually.

## 3. Results

### 3.1. Hoverfly numbers and biomass

**Figure 2.**
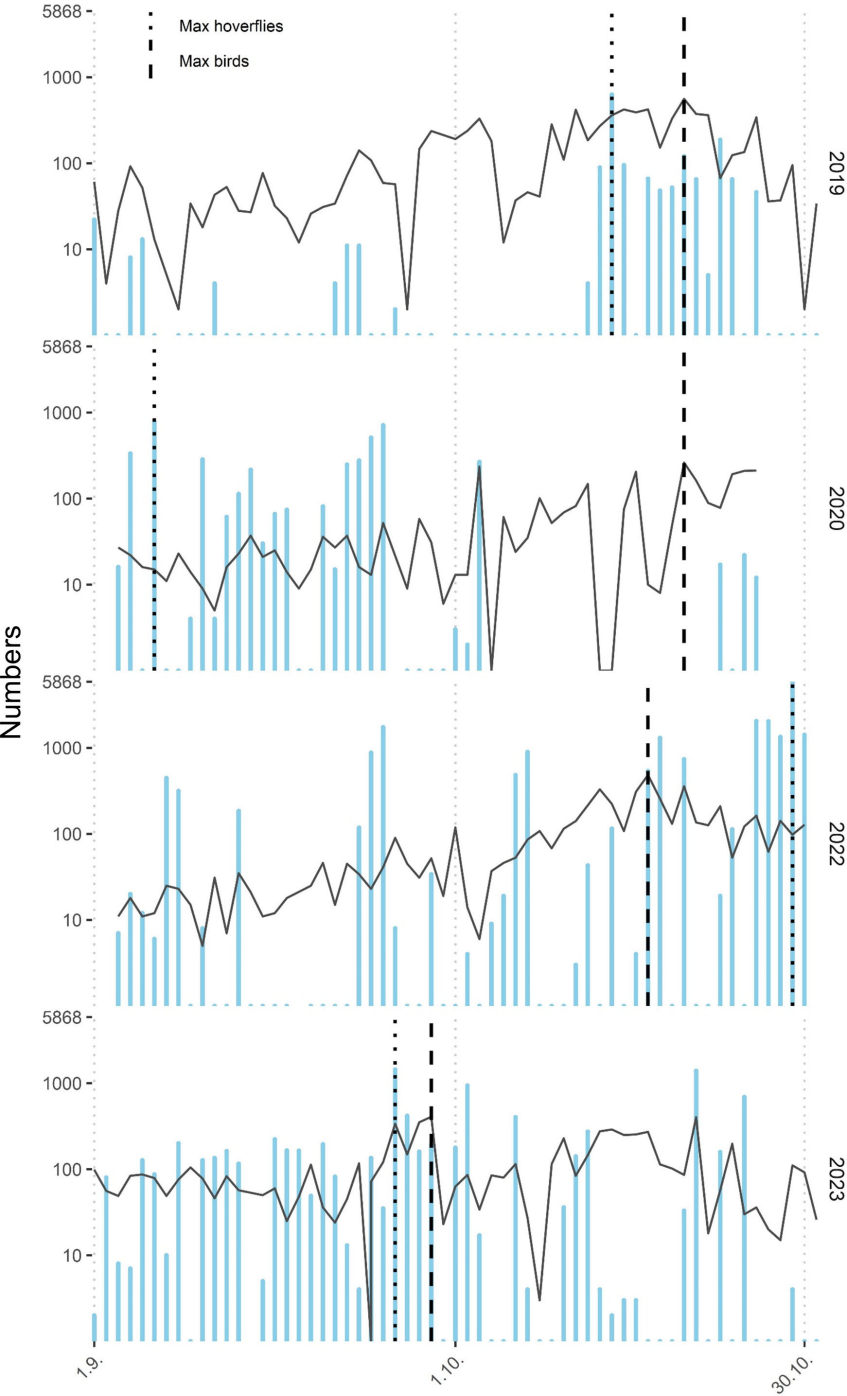
Total numbers of hoverflies (blue bars) and day-flying birds (black line) migrating southward yearly; *y*-axis is log-scaled. Maximum of hoverfly numbers (dotted line) and maximum of bird numbers (dashed line) are shown.

Overall, 35 087 migrating hoverflies of 47 species were recorded during 4 seasons. Their numbers strongly fluctuated between and within years, reaching up to 5868 hoverflies per day (figure 2). Five species represented the majority of the migrants: *Episyrphus balteatus* (De Geer, 1776) (64.73%), *Melanostoma mellinum* (L., 1758) (9.05%), *Eristalis tenax* (L., 1758) (6.42%), *Sphaerophoria* cf. *scripta* (L., 1758) (3.76%) and *Syrphus torvus* (F., 1794) (2.88%). For a complete list of migrating species, see electronic supplementary material, data.

**Figure 3.**
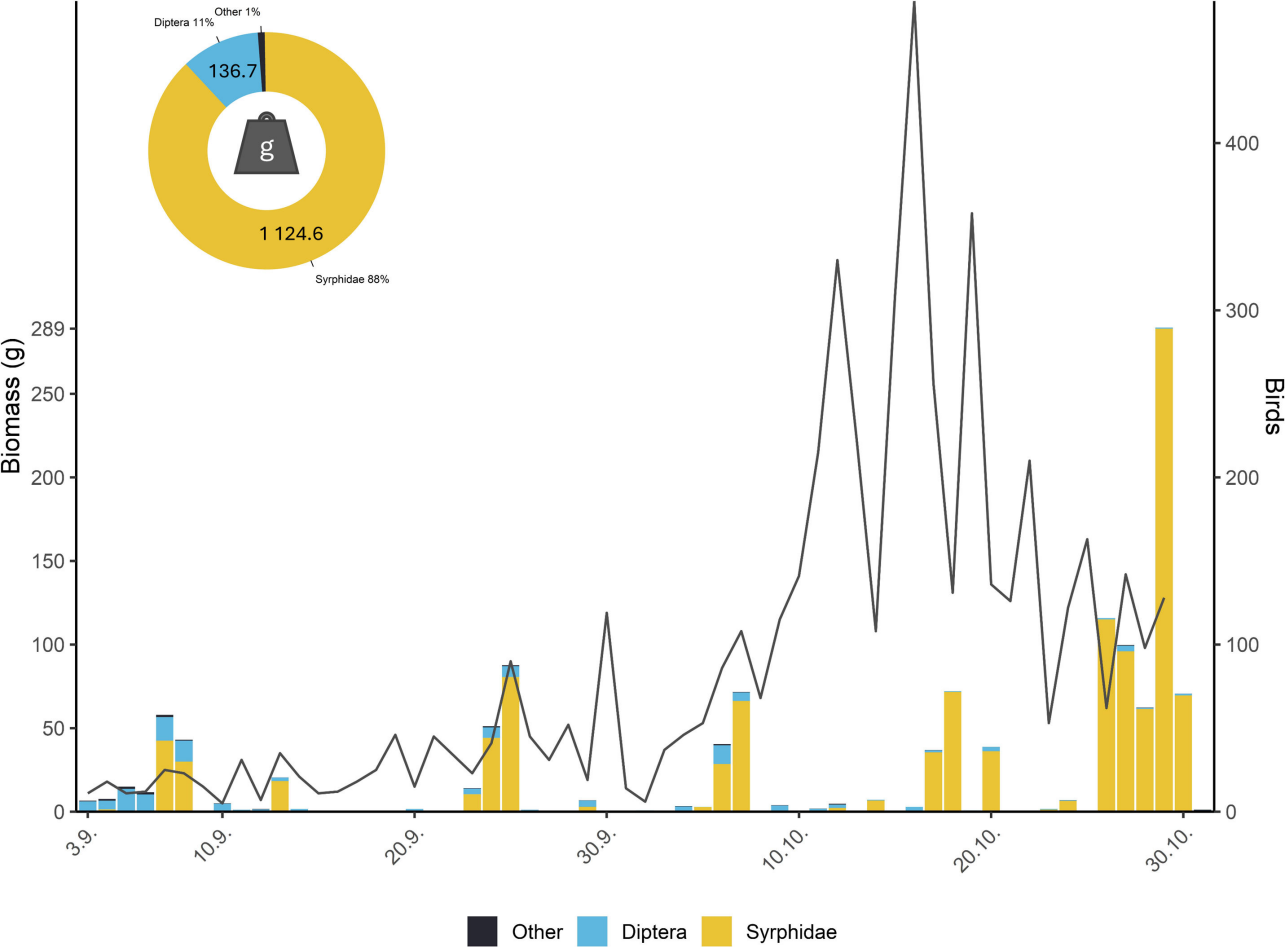
The proportion of biomass of day-flying insects during 2022: Syrphidae (yellow) accounted for 88% (1124.6 g), other dipterans (blue) for 11% (136.7 g) and different groups (black) as Hymenoptera, Hemiptera and Coleoptera for only 1% (12 g). The right-hand plot shows the biomass (colour bars) compared with day-flying bird numbers (black line).

Hoverfly biomass significantly outweighed that of the other groups. The total biomass of hoverflies was 1124.6 g (88% of total biomass), followed by other dipterans with 136.7 g (11%), Hymenoptera with 9.13 g (<1%), Hemiptera with 2.04 g (<1%) and Coleoptera with 0.83 g (<1%) (figure 3).

### 3.2. Bird community

We captured 23 094 individuals of 80 bird species (electronic supplementary material, data). Most bird species (67 species) were small passerines. Six most abundant species encompassing 78% of the whole sample were great tit (*Parus major*, 27.4%), goldcrest (*Regulus regulus*, 14.1%), dunnock (*Prunella modularis*, 10.4%), European robin (*Erithacus rubecula*, 9.6%), Eurasian bluet tit (*Cyanistes caeruleus*, 9.2%) and coal tit (*Periparus ater*, 8.1%). All these and the other 48 species sampled represent predominantly or strictly insectivorous species [46]. Only 0.8% (163 of 21 222) birds ringed in September and October between 2019 and 2022 were recaptured at the study site during the next 10 days following their initial capture. Daily totals of captured birds strongly correlated with numbers of birds observed migrating at the same site (*r*_spearman_ = 0.76, *n* = 211, *p* < 0.001; electronic supplementary material, figure S2).

### 3.3. Synchronization of bird and hoverfly migration intensity

We found that the number of birds caught in mist-nets was significantly positively correlated with the number of trap-caught hoverflies while controlling for a suite of confounding variables (table 1). The number of caught birds also increased with the Julian date and varied across years (table 1). The positive value in the first polynomial Julian date and the negative value in the second polynomial Julian date indicate that there is a unimodal association between the date and the strength of the migration, with the maximum in the middle of the autumn, decreasing to both early autumn and late autumn.

**Table 1.** Results of multiple linear regression with negative binomial distribution testing association between the number of caught birds in mist-nets (response variable) and the number of caught hoverflies, Julian date (second polynomial), year (reference level = 2019), wind speed and precipitations as predictors and covariates. Nagelkerke’s pseudo-*R* ^2^ of the model = 0.67. We report the slope (estimate) and its standard error (s.e.), *z*-values and *p*-values for each predictor. Statistically significant associations are highlighted in bold.

variable	estimate	s.e.	*z*-value	*p* **-**value
intercept	4.37131	0.18730	23.338	<0.001
**Julian date**	**8.79306**	**0.81337**	**10.811**	**<0.001**
**poly (Julian date)2**	**−1.82301**	**0.81915**	**−2.225**	**0.026**
**year 2020**	**−0.95786**	**0.18134**	**−5.282**	**<0.001**
**year 2022**	**−0.58592**	**0.15968**	**−3.669**	**<0.001**
year 2023	−0.05686	0.15615	−0.364	0.716
wind speed	0.04376	0.04913	0.891	0.373
precipitation	−0.09475	0.07415	−1.278	0.201
**hoverfly numbers**	**0.20051**	**0.06094**	**3.290**	**0.001**

Peaks of migration intensity for both birds and hoverflies took place at the end of October 2019 and 2022 and at the end of September 2023. In 2020, migration peaks of birds and hoverflies were decoupled; however, due to a storm, our sampling was interrupted for quite a long period (19 days), and we cannot exclude that peak migration took place during this period (figure 2).

## 4. Discussion

We found that the temporal patterns in the intensity of migration of day-migrating birds were strongly conjoined with that of hoverflies over Červenohorské sedlo mountain pass. In other words, we found that the number of birds caught by mist-netting was positively correlated, even after controlling for the effect of weather, with the number of hoverflies caught by traps, which indicates that birds and hoverflies co-migrate and that variation in intensity of migration may be driven by the same or similar factors. We also found that out of all flying insects passing through the studied mountain pass, hoverflies accounted for 88% of the total biomass. The migration of hoverflies is highly unexplored yet might be a keystone worldwide phenomenon [47], whereby tremendous movement of biomass, such as documented in Great Britain [24], might affect the local food webs and may serve as an invaluable pulse food source for both migratory and resident birds.

### 4.1. Temporal match in peaks of migration of birds and hoverflies

We found a statistically significant temporal overlap in the peaks of migration of birds and hoverflies at the study site. There are only a few documented examples of co-migration of hoverflies and their potential predators, such as dragonflies [48] and passerine birds [49,50]. However, the aforementioned studies are merely reports of incidental observations without the use of standardized methods to capture migrants. Furthermore, the time span of the former observations is limited to one or a few days. According to our best knowledge, our study is the first documented case of overlapping peaks in migration intensity in birds and hoverflies, using long-term and systematically collected data.

The studied site represents a typical barrier that both migrating birds and hoverflies overcome as quickly as possible, as the conditions in the mountains are unfavourable for them. Our data from recaptures of ringed birds clearly demonstrate that birds do not use the site for stopover, since only very low numbers (>1%) of birds have been recaptured at the study site during days following their initial capture. Based on these data and along with our long-term personal observations, there is no reason to expect that extensive trophic interactions between birds and syrphids occur directly at the site of our study.

However, the observed synchronization of the migrants may not take place only at a studied mountain locality but presumably occur at lowlands from which both groups ascended to the mountains as well as in lowlands where both groups were heading after they crossed this barrier. Ecological barriers, such as mountains, are costly to cross and can deplete energy reserves, and, consequently, birds are forced to stop and refuel shortly before or immediately after crossing a barrier [51]. Large quantities of the co-migrating hoverflies may thus provide a valuable source of energy for birds at their stopover sites. Moreover, it has been demonstrated that migratory hoverfly individuals are less active than sedentary ones [52], potentially being even more attractive prey to migrating birds than their non-migratory relatives. This topic deserves further research since no study exists, to our knowledge, on the role of hoverflies in birds’ diet during autumn migration.

### 4.2. Hoverfly numbers and insect biomass

Our study also documents the extent of the autumnal hoverfly migration and their biomass compared with other day-flying insects over a mountain pass in Central Europe. Migrating hoverflies are the most abundant food source passing through Červenohorské sedlo, accounting for 88% of the total biomass in 2022. We found a much higher proportion of hoverflies compared with other migrating insects than reported by previous studies [25,53] on spring migration in Cyprus (hoverflies represented 17% of all insect migrants) and autumnal migration through Puerto de Bujaruelo in Spain, where syrphids encompassed 20% of all migratory insects. These differences might be attributed to disparity in methods and metrics used. We note that we estimated hoverfly biomass, not the numbers of insects other than hoverflies. However, since biomass and numbers are strongly correlated, we can make this comparison [54]. The differences in the ratios of certain taxa, such as Rhopalocera, observed between our site and those in Cyprus and Spain can be attributed to the different methodologies used, as the Malaise trap has several inherent limitations (a detailed discussion of which is provided in §4.4). However, we recorded noticeably less of other Diptera (excluding hoverflies). The results suggest that species composition of the autumnal and spring migration is quite different between these sites and probably also differs along a latitudinal gradient.

The species composition of migrating hoverflies was similar to that of previous studies, but the proportion of species varied. Aubert *et al*. observed autumnal migration of hoverflies through Col de Bretolet from 1962 to 1973 [55,56]. The most predominant species in their studies were *E. balteatus* (52.8 %) and *E. tenax* (26.5%). Similarly, Gatter and Schmid in observations from Randecker Maar [57,58], lasting for 40 years, reported as the most abundant hoverflies *E. balteatus* (33.2%) and *Platycheirus clypeatus*/*peltatus* (17.7%) from 1978 to 1987, and *M. mellinum* (23%) and *E. balteatus* (22.8%) from 2015 to 2019. This shift in composition can be caused by several factors, one of which may be the loss of native habitats and landscape fragmentation, resulting in the loss of formerly common species or a decrease in population size and abundance [54,59].

### 4.3. Weather conditions

In our study system, precipitation negatively affected hoverfly migration, while birds migrated regardless of the weather conditions. The influence of weather on bird migration is known to be strongly species-specific [60,61], and thus its effects might be blurred when individuals of all migrating species are analysed together. Hoverfly migration is known to be influenced by a range of weather conditions, such as precipitation, wind direction and speed, temperature or barometric pressure [35,62,63]. In our analysis, weather conditions appeared to have only a relatively weak effect on migration of hoverflies and birds. Our findings seemingly contrast with previous studies, even ours from the same study site, where we also observed a positive effect of mean daily temperature and wind direction on hoverfly migration [35]. A limitation of our study is the scarcity of northerly winds (in day mean) at the Červenohorské sedlo [35], making it nearly impossible to assess the effect of wind direction on migration intensity. Moreover, the effects of wind direction or temperature on the migration of birds and hoverflies were masked by other covariates, such as date or precipitation.

### 4.4. Limitations

The primary source of unreliability is the method used to monitor insect migration. Already Malaise noted that this type of trap is very place sensitive [64]. In addition, Malaise trap efficiency differs for various orders (with highest efficiency for Hymenoptera and Diptera) [65] and even for species within families [66,67]. Therefore, since the beginning of the project in 2018, we have caught only a handful of butterflies and dragonflies, which are otherwise common migrants [68,69]. Further data collection, using other types of traps or monitoring methods, such as standardized visual observation [25,53], would be needed to determine precisely what proportion of migrants is formed by groups undetectable by the Malaise trap.

Another important limitation is the lack of direct data on the diet of migrating birds not only at our study site. While most of the recorded bird species are predominantly insectivorous, the extent to which hoverflies constitute a significant part of their diet remains unknown. Future studies incorporating faecal analysis (e.g. using metabarcoding) or stable isotope analysis could provide more details on prey selection by birds during migration and help clarify the importance of hoverflies in the diet of migratory birds.

A significant source of uncertainty relates to the influence of endogenous rhythms and life-history traits that may vary across species and animal clades on the observed migration patterns of birds and hoverflies. The timing of migration in birds is driven by species-specific life-history traits, such as reproduction, moulting timing and physiological changes, as well as by environmental cues like photoperiod, which regulate endogenous rhythms in both daily behaviours, including migratory restlessness (Zugunruhe), and broader annual cycles essential for migration [70–72]. The drivers of onset of hoverfly migration are much less studied than those in birds, but at least some of them apply also for hoverflies. For instance, hoverflies have well-documented migratory behaviours that are tied to seasonal reproductive cycles and environmental cues [73,74]. Whereas similarity in the drivers of migration timing may reinforce co-migration across bird and hoverfly species, differences may disrupt these patterns, calling for studies on broader taxonomic scales.

While our analysis accounts for Julian date as a temporal variable, this may not fully capture the complex interactions between species-specific traits and environmental conditions. For example, Julian date partially captures seasonal variation but does not distinguish between early-migrating and late-migrating species or account for year-to-year variability in migration cues, such as temperature or food availability at breeding grounds. Further refinement of analysis to incorporate species-specific phenological data would improve our understanding of the temporal dynamics observed in bird and hoverfly migration.

The use of acoustic lures to capture birds is known to influence both the efficiency and species composition of captures. Moreover, this method may lead to biased sex ratios in some species [75,76]. However, our research aimed to monitor changes in migration intensity, rather than to examine the exact proportions of species. The use of acoustic lures attracted some of the birds flying over the installed mist-nets, thereby significantly increasing the effectiveness of our sampling. Since the same bird call recordings were used each year, there is no reason to believe this method affected the observed variation in migration intensity. This is also supported by the fact that numbers of captured birds highly correlated with numbers of birds observed visually (electronic supplementary material, figure S2). Altogether, daily bird capture totals well corresponded with the real intensity of bird migration at our study site.

Finally, we must acknowledge that our dataset is geographically limited to a single mountain pass in Central Europe. The patterns we observed may not be generalizable to other migration corridors, particularly those in different climatic regions or with different ecological barriers. Long-term monitoring at additional sites will be essential to fully understand the broader implications of our findings.

## 5. Conclusion

In this study, we have revealed the temporal synchronization between hoverfly and bird migration. Based on a four-year study of autumn migration through the Jeseníky Mountains, we found a strong correlation between bird and hoverfly numbers. Hoverflies accounted for 88% of daytime-flying insect migrants at the site. Taken together, these findings suggest that hoverflies may play a significant role in the diet of both migrating and resident insectivorous birds. This study is the first step towards enhancing our understanding of insectivorous birds and their prey co-migration. We hope that our research will serve as a base for future studies on bird–insect co-migration, particularly concerning hoverflies.

## Data Availability

The R code and data are provided as electronic supplementary material [77].
